# The influence of COVID-19 preventive measures on the air quality in Abu Dhabi (United Arab Emirates)

**DOI:** 10.1007/s11869-021-01000-2

**Published:** 2021-04-04

**Authors:** Oriol Teixidó, Aurelio Tobías, Jordi Massagué, Ruqaya Mohamed, Rashed Ekaabi, Hussein I. Hamed, Richard Perry, Xavier Querol, Shaikha Al Hosani

**Affiliations:** 1 Environment Agency – Abu Dhabi (EAD), Abu Dhabi, United Arab Emirates; 2 Institute of Environmental Assessment and Water Research (IDAEA), Spanish Council for Scientific Research (CSIC), Barcelona, Spain

**Keywords:** SARS-CoV-2, COVID-19, Lockdown, Air pollution, Air quality, UAE

## Abstract

The preventive and cautionary measures taken by the UAE and Abu Dhabi governments to reduce the spread of the coronavirus disease (COVID-19) and promote social distancing have led to a reduction of mobility and a modification of economic and social activities. This paper provides statistical analysis of the air quality data monitored by the Environment Agency – Abu Dhabi (EAD) during the first 10 months of 2020, comparing the different stages of the preventive measures. Ground monitoring data is compared with satellite images and mobility indicators. The study shows a drastic decrease during lockdown in the concentration of the gaseous pollutants analysed (NO_2_, SO_2_, CO, and C_6_H_6_) that aligns with the results reported in other international cities and metropolitan areas. However, particulate matter (PM_10_ and PM_2.5_) averaged concentrations followed a markedly different trend from the gaseous pollutants, indicating a larger influence from natural events (sand and dust storms) and other anthropogenic sources. The ozone (O_3_) levels increased during the lockdown, showing the complexity of O_3_ formation. The end of lockdown led to an increase of the mobility and the air pollution; however, air pollutant concentrations remained in lower levels than during the same period of 2019. The results in this study show the large impact of human activities on the quality of air and present an opportunity for policymakers and decision-makers to design stimulus packages to overcome the economic slow-down, with strategies to accelerate the transition to resilient, low-emission economies and societies more connected to the nature that protect human health and the environment.

**Supplementary Information:**

The online version contains supplementary material available at 10.1007/s11869-021-01000-2.

## Introduction

On 11 March 2020, the World Health Organization (WHO) announced that the COVID-19 outbreak was characterised as a pandemic and urged countries to take urgent and aggressive action (World Health Organization 2020a). According to statistics, as of 6 November 2020, the cumulative number of confirmed cases of COVID-19 has exceeded 48 million (World Health Organization 2020b).

In response to the global outbreak, United Arab Emirates (UAE) public authorities took proactive actions to protect the safety and well-being of citizens, residents, and visitors. The preventive and cautionary measures that were taken by the UAE and Abu Dhabi governments to reduce the spread of the virus SARS-CoV-2 and promote social distancing led to reduced mobility and modification of economic and social activities.

The Ministry of Education announced that spring break for students would start on 8 March until 19 March, and distance learning started on 22 March (UAE Ministry of Education 2020). Abu Dhabi government activated the remote work systems for government employees on 12 March for vulnerable populations and on 24 March for 100% of the workforce. For the private sector, a maximum of 30% of the workforce was allowed to be physically present, except for the critical sectors, such as health, pharmaceuticals, energy, and water. On 26 March, the UAE National Disinfection Programme started, with daily disinfection, from 8 pm to 6 am. Movement of traffic and people during the disinfection period was restricted, except for food and pharmaceutical needs and critical sectors. Passenger flights were suspended on 25 March to protect citizens, residents, and international travellers. On 24 June, the UAE government announced the completion of the National Disinfection Programme, including the night curfew; however, several preventive measures remained active such as ban on public gatherings, maintaining social distancing norms, and controls in the entry borders of Abu Dhabi Emirate.

Similar lockdown measures have been taken in other countries worldwide resulting in a reduction of the air pollution (Tobías et al. 2020, Nakada and Urban 2020, Dantas et al. 2020, Sharma et al. 2020, Wang and Su 2020, Wang et al. 2020, Mahato et al. 2020, Broomandi et al. 2020, Kerimray et al. 2020, Otmani et al. 2020, Sicard et al. 2020, among others). Analysis performed at cross-national level also indicates a significant decrease in global air pollution (Dang and Trinh 2020, Venter et al. 2020). However, to our knowledge, there is still little evidence published in the countries of the Gulf Cooperation Council (GCC). We only found a recently published study of Eastern Province, Saudi Arabia, reporting a similar reduction of air pollution levels compared to other regions worldwide (Anil and Alagha 2020).

The aim of this study is to assess the changes in air quality during and after the implementation of the preventive measures implemented to reduce the spread of the COVID-19 epidemic in Abu Dhabi (UAE) and learn how air quality can be improved by decreasing anthropogenic emissions in this region. The study also includes the assessment of the changes in the meteorological and mobility data available during the same periods.

## Method

### Study area

The United Arab Emirates (UAE) is a federation of seven emirates founded in 1971. The largest of the emirates, Abu Dhabi, covers around 67 000 km^2^, accounting for 87% of the area of the UAE, and is home to 2.9 million inhabitants, almost 30% of the UAE population (SCAD 2020; Environment Agency - Abu Dhabi 2017). Abu Dhabi Emirate is divided into three regions: Abu Dhabi Region, Al Ain Region, and Al Dhafra Region. Abu Dhabi city, in the Emirate of Abu Dhabi, is the capital of the UAE. Emissions from industry and transport, as well as desert dust events, are the main pressure on air quality in the emirate (Environment Agency - Abu Dhabi 2017).

### Air quality and meteorology data

Environment Agency – Abu Dhabi (EAD) monitors air quality in the emirate through an integrated network of 20 fixed and 2 mobile stations. The network monitors up to 17 parameters using the latest technologies and methods and adheres to the testing and competency standards of ISO/IEC 17025:2017. Selected meteorological parameters are measured in all stations. Minute-based monitoring data from the network stations are transferred automatically to a central database continuously using an up-to-date Air Quality Management System. The data is continuously stored in the EAD database for quality check, control, evaluation, validation, statistical treatment, and presentation on a web portal. Quality-controlled data are then stored in the final database for further analysis. The Air Quality Monitoring programme in Abu Dhabi fulfils the conformity aspects regarding the measurement methods, as the instrument principles used are all in accordance with ISO, CEN/EN, and US standards. Traceability is ensured using traceable gas calibration standards. Maps with location of the EAD air quality monitoring stations and live data broadcasts on the status of air quality are available on the EAD Air Quality website (https://www.adairquality.ae/).

This study analyses the air quality data from Abu Dhabi Region, accounting for 62.1% of the population in the emirate (SCAD 2020). EAD operates 8 fixed stations in Abu Dhabi Region, covering different area types, as described in Table S1. All validated hourly values from the 8 stations have been considered to calculate the daily average concentration levels of nitrogen dioxide (NO_2_), sulphur dioxide (SO_2_), carbon monoxide (CO), benzene (C_6_H_6_), and particulate matter (PM) with a diameter of less than 10 μm (PM_10_) and 2.5 μm (PM_2.5_). In the case of ozone (O_3_), the daily maximum 8-h means have been used in order to take into account the increased health effects of exceeding the WHO guideline value based on daily maximum 8-h mean (WHO 2006). The meteorological data from all EAD stations have been used following the same methods. Data for the same period in 2019 (1 January until 24 October) has been retrieved for the same stations to assess the year-to-year variation in the mean and relative change.

The PM_2.5_/PM_10_ ratio has been calculated to assist in the evaluation of the influence of desert dust episodes in the variation of PM_2.5_ and PM_10_ during the different study periods. As described by Querol et al. 2019, the ratio PM_2.5_/PM_10_ will provide the load of fine particles that in part might be attributable to anthropogenic pollution. However, PM_2.5_/PM_10_ ratios in pure mineral dust might also vary as a function of the source area and the transport pathways and duration. Most of the anthropogenic PM pollution falls within the PM_2.5_ fraction, and if increased, it will increase the PM_2.5_/PM_10_ ratio.

Finally, remote sensing NO_2_ data, measured by the Copernicus Sentinel-5 Precursor Tropospheric Monitoring Instrument (S5p/TROPOMI) developed by the European Space Agency (ESA), has been used to assess tropospheric NO_2_ background levels in a high resolution (3.5×5.5 km^2^) continuous area (Veefkind et al. 2012). To this end, a script has been written to retrieve, calculate mean levels, and plot over a map the NO_2_ data using Google Earth Engine (Gorelick et al. 2017).

### Mobility data

‘COVID-19 Community Mobility Reports’ were prepared by Google in 2020 (Google 2020). A daily time series data was gathered using location and highlights the percentage of changes for each day from the baseline value. Data collection started from 15 February 2020 and includes before, during, and after the COVID-19 lockdown period in different regions of the World. A baseline day represents a normal value for that day of the week and is estimated as the median value from the 5-week period, 3 January to 6 February 2020. The reports present the mobility trends according to different categories: (i) retail and recreational mobility, (ii) grocery and pharmacy mobility, (iii) parks mobility, (iv) transit stations mobility, (v) workplaces mobility, and (vi) residential mobility (Google 2020).

For this study, data is used from ‘Google COVID-19 Community Mobility Reports’ for Abu Dhabi Emirate. Data was downloaded in CSV format and processed to evaluate the mobility trends in Abu Dhabi according to the study periods. The data used in this study is available in the public domain (https://www.google.com/covid19/mobility/).

### Study period

The study analyses almost 10 months of data. Three study periods have been designed based on the preventive measures in place and the latest data available: (i) pre-lockdown (1 January to 21 March 2020) is used as the baseline for the analysis; (ii) lockdown (22 March to 24 June 2020); and (iii) post-lockdown (25 June to 24 October 2020).

### Statistical analysis and data visualisation

Descriptive statistics were used to describe daily average concentrations of each pollutant assessing the variation in the mean concentration (μg/m^3^) and their relative change (%), before, during, and after the lockdown in 2020. Linear regression models have been used to test for differences on daily average concentrations of each pollutant during and after the lockdown in comparison with the pre-lockdown period, except for ozone, where daily maximum 8-h means have been used. Time trends have been plotted for the averaged values in the region and for the individual stations (in grey) to evaluate common trends and differences among stations. The weekly averages have been plotted to avoid the weekend effect in the visual evaluation of the trend results (Blanchard and Tanenbaum 2003). Same methods have been used to test for differences on available meteorological parameters from Abu Dhabi region stations: wind speed, temperature, and net radiation. NO_2_ hourly statistical analysis of the mean and 95 % confidence interval has been performed for an urban traffic station to assess the impact of the night curfew. Additionally, comparison with 2019 data has been done for the same stations and study period, including comparison of the 7-day rolling average concentrations for the available pollutants.

All mobility data obtained from ‘Google COVID-19 Community Mobility Reports’ for Abu Dhabi Emirate has been used. The average of all the categories has been calculated to facilitate the evaluation and presentation of the results. The same statistical methods used for the air pollutants have been applied to the mobility indicator.

All the air quality, meteorology and mobility data were imported and processed using the R version 3.6.0 language and environment (R Core Team 2019).

## Results

### Meteorology comparison

Meteorological conditions during the study period showed a significant increase of the mean temperature from 21.0 °C before the lockdown up to 31.0 and 34.7 °C during and after the lockdown, respectively (Table S2). The increase was also evidenced for the insolation ranging the net radiation from 133.5 W/m^2^ before the lockdown to 151.0 and 151.3 W/m^2^ during and after lockdown, respectively. Moreover, wind speed values remained similar in all periods between 1.9 and 2.1 m/s with no significant variations compared to the baseline (*p*=0.029 and *p*=0.254).

### Air pollution variation during study periods

**Fig. 1:**
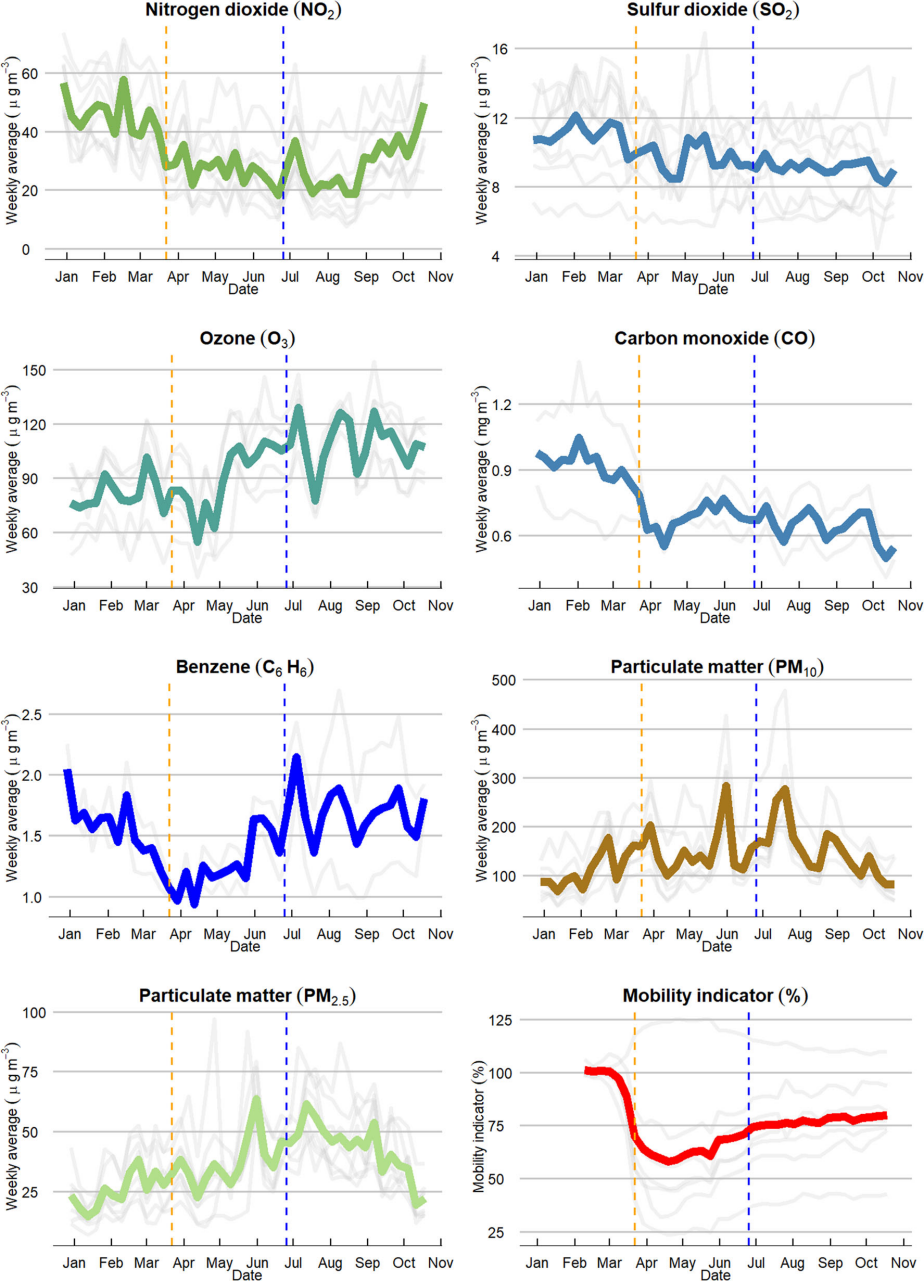
Average concentrations of nitrogen dioxide (NO_2_), sulphur dioxide (SO_2_), ozone (O_3_), carbon monoxide (CO), benzene (C_6_H_6_), particulate matter (PM_10_), and particulate matter (PM_2.5_) between 1 January 2020 and 24 October 2020 (with lockdown starting on 22 March 2020 and ending on 24June 2020) in Abu Dhabi, UAE (in grey are drawn the averaged values for each station monitoring that pollutant). In the lower right of the figure: average of the Google Mobility indicator compared to baseline mobility (in grey the average values by mobility category)

During the lockdown period, air pollution decreased with substantial differences among pollutants compared to the baseline (Fig. 1). A significative decrease of NO_2_ concentrations was experienced, with a −40 % reduction equivalent to −18.1μg/m^3^ on average (Table 1). Similarly, SO_2_, CO, and C_6_H_6_ reductions were significant, with a decrease of −12.2, −25.8, and −19.9%, respectively. O_3_ experienced a decrease during April (Fig. 1), but rapidly increased during the next months, with an average increase of +17.0% during the lockdown period compared to 2020 baseline. PM_10_ and PM_2.5_ underwent significant increases of +33.4 and +45.0%, respectively.

**Table 1:** Average concentrations of NO_2_, SO_2_, O_3_, CO, C_6_H_6_, PM_10_, and PM_2.5_ between 1 January to 21 March 2020 (pre-lockdown), 22 March to 24 June 2020 (during the lockdown), 25 June to 24 October 2020 (after lockdown), and differences with pre-lockdown period in Abu Dhabi, UAE

	Pre-lockdown	Lockdown				Post-lockdown			
	Average (μg/m^3^)	Average (μg/m^3^)	Difference			Average (μg/m^3^)		Difference	
			μg/m^3^	%	*p*-value		μg/m^3^	%	*p*-value
Nitrogen dioxide (NO_2_)	45.3	27.2	−18.1	−40.0	<0.001	29.1	−16.2	−35.8	<0.001
Sulphur dioxide (SO_2_)	11.1	9.7	−1.4	−12.2	<0.001	9.1	−2.0	−17.6	<0.001
Ozone (O_3_)*	56.2	65.8	9.6	+17.0	0.001	75.2	19.0	+33.8	<0.001
Carbon monoxide (CO)	0.9	0.7	−0.2	−25.8	<0.001	0.6	−0.3	−31.0	<0.001
Benzene (C_6_H_6_)	1.6	1.3	−0.3	−19.9	<0.001	1.7	0.1	+8.4	0.007
Particulate matter (PM_10_)	112.3	149.9	37.6	+33.4	0.001	151.9	39.6	+35.2	0.001
Particulate matter (PM_2.5_)	25.3	36.7	11.4	+45.0	<0.001	43.3	18.0	+71.1	<0.001

*Ozone daily averages of the daily maximum 8-h mean

After the lockdown ended, the NO_2_ concentrations experienced an increase during the first weeks of July but remained in lower levels during August. NO_2_ experienced a steady increase from September until the end of October (latest available data). The average NO_2_ concentrations after lockdown are −35.8% lower than before lockdown and +7% higher than during lockdown. SO_2_ and CO concentrations after lockdown continued in the same levels as during lockdown and even reduced the average concentration by −6 % and −7 %, respectively. C_6_H_6_ had strong variations during the post-lockdown with an average increase of +35 % compared to the lockdown period. O_3_ continued the increase during the post-lockdown with an additional +14 %. After the lockdown, PM_10_ and PM_2.5_ underwent strong week-to-week variations, but with an overall significant increase compared to pre-lockdown period.

The air pollution changes during the study periods are consistent among the air quality stations analysed, represented by the grey lines in Fig. 1. Some stations monitored higher levels of SO_2_, PM_10_, and PM_2.5_ in May and June. Hourly and minute data was analysed, and local experts consulted to determine that the increases were due to local events, such as maintenance activities in industrial and oil and gas facilities and construction activities.

**Fig. 2:**
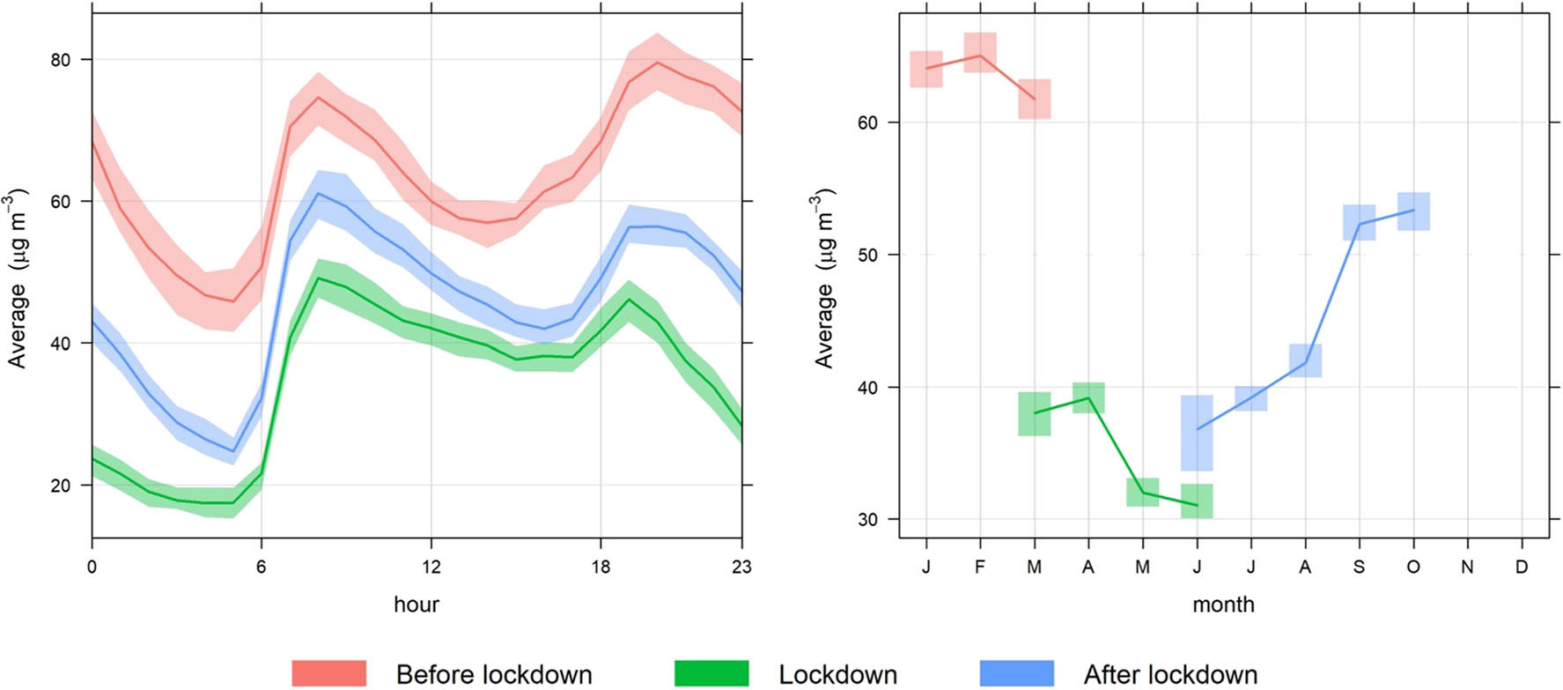
Hourly and monthly variation of NO_2_ in μg/m^3^ and 95% confidence intervals during the three defined periods of 2020 in the Hamdan air quality monitoring station (urban traffic station) in Abu Dhabi, UAE

Comparing the hourly air pollution levels for the three periods in an urban traffic station, it is observed that during lockdown, the NO_2_ concentrations were markedly lower than before and after lockdown periods (Fig. 2). Furthermore, the largest reductions were observed after 8 pm until 6 am, when the night curfew was in place and traffic and movement restrictions were imposed.

**Fig. 3:**
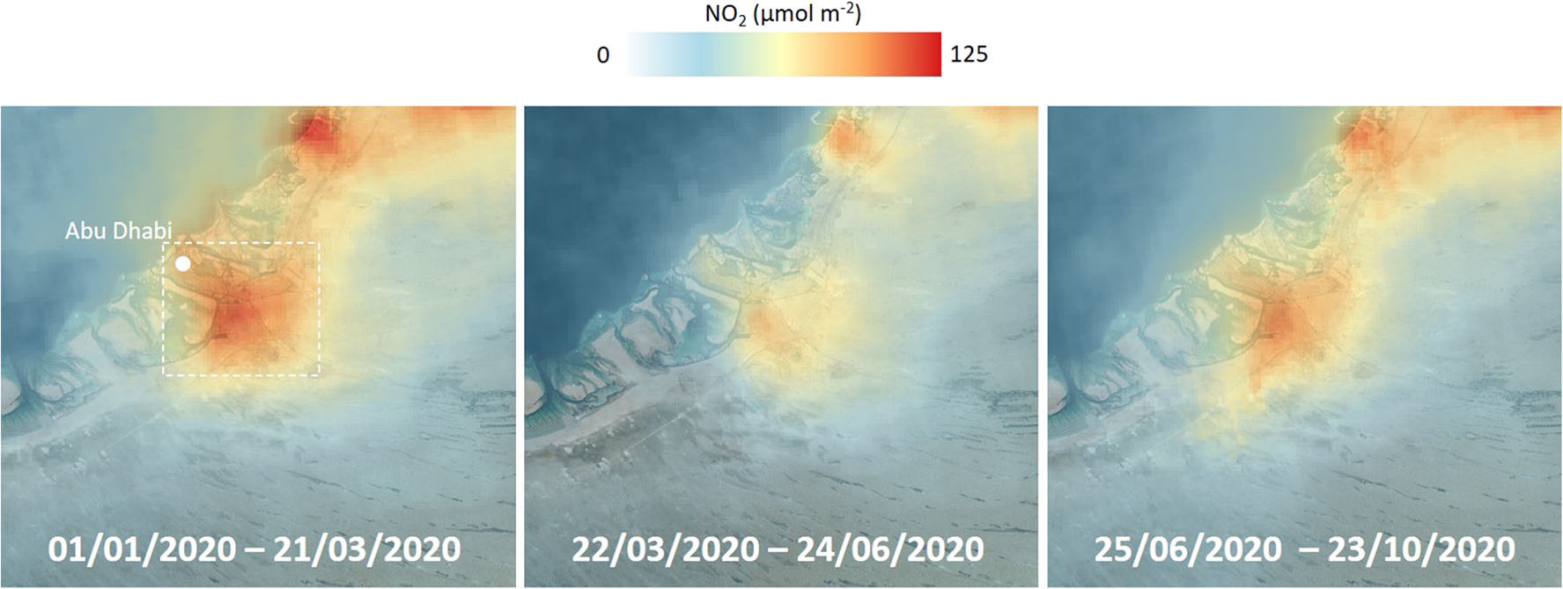
Average levels of background tropospheric NO_2_ measured by TROPOMI-ESA in the Arabian Peninsula during the pre-lockdown period and the lockdown period (both mild lockdown and hard lockdown considered together). Dotted lines around Abu Dhabi mark the area used to quantify the NO_2_

The major changes for NO_2_ are also clearly shown by satellite measurements of background tropospheric NO_2_ concentrations supplied by TROPOMI-ESA. Averaged TROPOMI NO_2_ levels over Abu Dhabi decreased −37% during the lockdown and −13% after lockdown compared with the pre-lockdown levels, both reductions consistent with the surface measurements (Fig. 3).

### Comparison with 2019 data

**Fig. 4:**
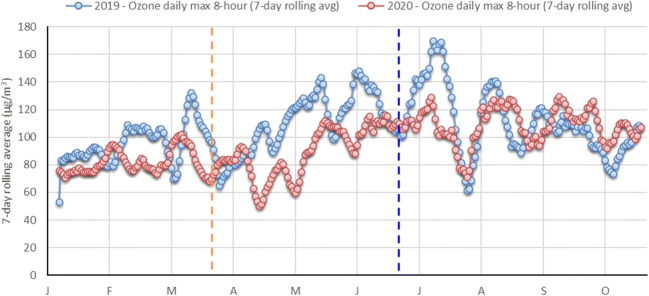
Ozone daily maximum 8-h mean for the same period in 2019 and 2020 (orange dotted line indicates the start of the lockdown on 22 March 2020 and blue dotted line indicates the end on 25 June 2020) in Abu Dhabi

The averaged concentrations during lockdown and those for the same period in 2019 also show the largest decrease for NO_2_, with a −40 % reduction (Table 2). Reductions are also significant for SO_2_, O_3_, and CO, with an average reduction of −18%, −15%, and −21%, respectively. Benzene data was not available for 2019, and therefore the comparison was not possible. PM_10_ averaged concentrations monitored during the lockdown in 2020 were higher than for the same period in 2019 (+26 %), and PM_2.5_ concentrations were a −9% lower, pointing to a large impact of desert dust events during the 2020 lockdown period in Abu Dhabi. Thus, in 2019, the PM_2.5_/PM_10_ ratio reached 0.28, 0.34, and 0.37 in the pre-lockdown and post-lockdown periods, respectively. The low PM_2.5_/PM_10_ ratios are typical of regions with high resuspension of dust (Querol et al. 2019), but the decrease down to 0.24 during the 2020 lockdown period indicates a higher desert dust influence. Ozone daily maximum 8-h means remained below the 2019 values (Table 2, Fig. 4).

**Table 2:** Relative variation of NO_2_, SO_2_, O_3_, CO, PM_10_, and PM_2.5_ between 2020 vs 2019 for the same study periods in Abu Dhabi, UAE

	NO_2_	SO_2_	O_3_	CO	PM_10_	PM_2.5_
1 Jan until 21 March	1%	26%	−19%	4%	7%	−13%
22 March until 24 June	−40%	−18%	−15%	−21%	26%	−9%
25 June until 23 October	−6%	−12%	0%	−41%	−2%	2%

### Mobility trends during study periods

**Fig. 5:**
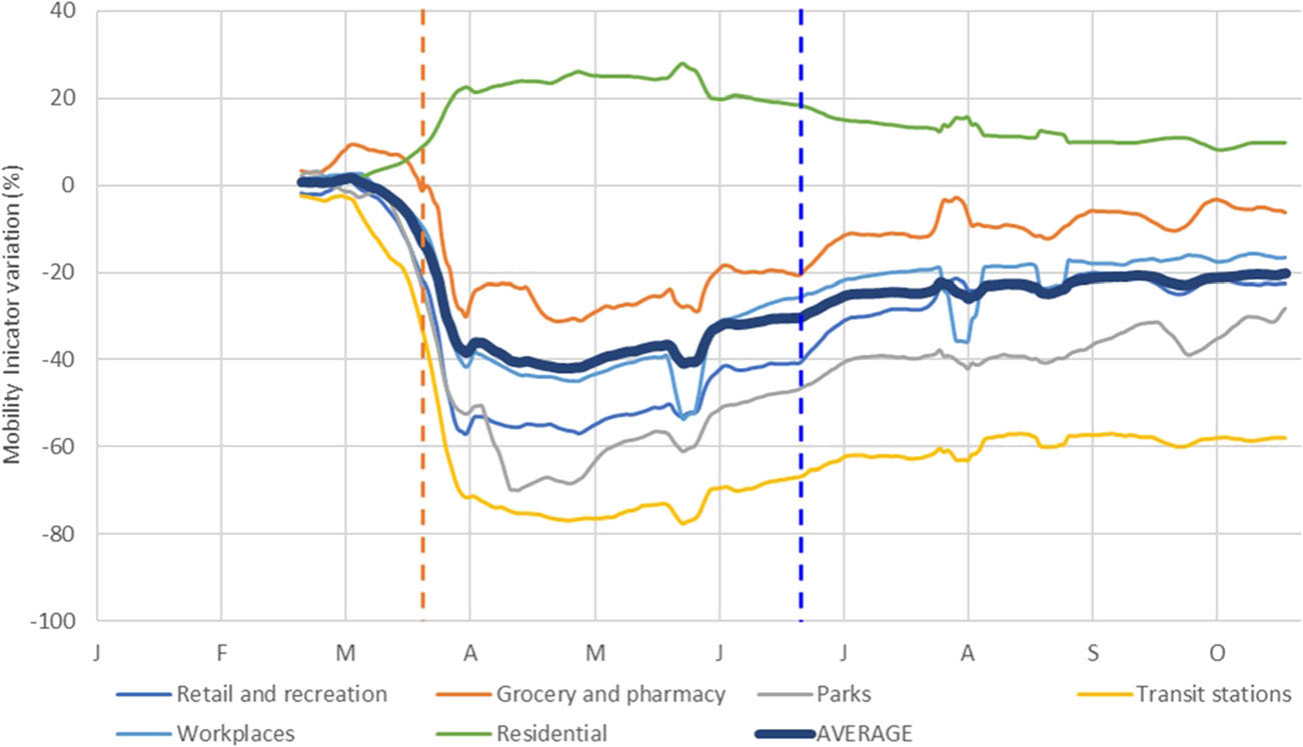
7-day rolling average of the mobility indicator compared to baseline for the different categories between 15 February 2020 and 24 October 2020 (blue dotted line marks the start of the lockdown on 22 March 2020 and orange dotted line marks the end on 25 June 2020) in Abu Dhabi

The mobility trends of Google COVID-19 Community Mobility Reports show a drastic reduction of the mobility when the strict lockdown measures were implemented in mid-March (Table S1, Fig. 5). All the categories show a similar pattern, except residential, which increased up to 20 percentual points. This growth is due to the increase in the amount of time spent at places of residence, and similar increases have been reported in different parts of the world (Google 2020; Saha et al. 2020).

The largest decreases were in the transit stations, parks, and retail and recreation categories, with an averaged reduction compared to baseline of −60%, −52%, and −44%, respectively. In workplaces category, the mobility reduction during lockdown has been measured as −37 % according to the Google Mobility reports and −29% in grocery and pharmacy category.

After the stricter preventive measures were lifted on 24 June, the mobility slowly increased but has not yet reached the same conditions as before the lockdown. Transit stations, which include public transport, remains the category with the highest reduction, with an average of −48% compared to before lockdown values. The workplaces category significantly increased after lockdown reaching an average of −20% compared to baseline.

## Discussion

NO_2_ had the largest and most significant reduction amongst the pollutants monitored (−40% comparing the lockdown period with the pre-lockdown values and −40% comparing 2020 vs*.* 2019 data). NO_2_ is mainly emitted from combustion processes, mostly road traffic in urban areas. Other key emitting sectors in Abu Dhabi are electricity generation, oil and gas activities, industry, shipping, and aviation (Environment Agency – Abu Dhabi 2018). The most substantial reductions have been observed during night-time when the National Sterilisation Programme was in place, where there was a mandatory stay-at-home policy in place, reducing people’s movement and traffic. The average reduction of NO_2_ is possibly related to the large drop in mobility during the same period, −36.2% on average during lockdown and −22.9% post-lockdown. CO and C_6_H_6_ had similar reductions and patterns during the lockdown period, indicating a potential common source of emissions. Both pollutants are considerably influenced by road transport emissions in urban environments. Ozone is a secondary pollutant formed in the atmosphere by the reaction of NOx and volatile organic compounds (VOCs) in the presence of sunlight (Monks et al. 2015, and references therein). An overall increase in O_3_ has been observed during lockdown and post-lockdown, +17.0% and +33.8%, respectively; however, daily maximum concentrations remained below the 2019 values. Even though these increases may seem counterintuitive, it is a consequence of the complex O_3_ chemical formation process. Both insolation and temperature increased in the lockdown and post-lockdown compared with the pre-lockdown, and this might have enhanced the O_3_ formation potential. Similar increases have been reported in Barcelona (Tobías et al. 2020) and India (Sharma et al. 2020) during the COVID-19 lockdowns. Sicard et al. (2020) studied O_3_ trends in three European cities and one Chinese city and concluded that the lockdown effect on O_3_ production was higher than the weekend effect, mainly due to a reduction in NO_x_ emissions from road traffic leading to a lower O_3_ titration by NO. Further research is recommended to understand the complex dynamics of O_3_ formation during extended periods of reductions in precursor emissions.

Particulate matter concentrations show a very different pattern from the rest of pollutants analysed and with substantial week-to-week variations. Dust events are frequent in the Arabian Peninsula (Otaibi et al. 2019), and the PM_10_ and PM_2.5_ averaged concentrations have been more impacted by desert dust episodes than the preventive measures implemented. The comparison of the PM_2.5_/PM_10_ ratio between 2019 and 2020 during lockdown period indicated a higher dust influence in 2020. Further research is required to understand the influence of local emissions and regional dust events during this period.

The result observed in Abu Dhabi is consistent with the recent literature. Tobías et al. (2020) reported reductions of pollutants mainly related to traffic emissions, NO_2_ and black carbon (BC), of −51% and −45%, during the lockdown period compared to before the lockdown in the city of Barcelona (Spain). Nakada and Urban (2020) reported drastic reductions of NO (up to −77.3%), NO_2_ (up to −54.3%), and CO (up to −64.8%) in São Paulo (Brazil) during partial lockdown compared to the 5-year monthly mean. Similar results were also found in Rio de Janeiro with reductions of NO_2_ (37.0 to 43.6%) and CO (21.4 to 32.9%) (Dantas et al. 2020). In India, the air quality index decreased by 44, 33, 29, 15, and 32% in north, south, east, central, and western regions, respectively, due to the effect of restricted human activities during the COVID-19 pandemic (Sharma et al. 2020). While a study conducted in China using satellite data stated that the strict quarantine measures led to a reduction in NO_2_ emissions (Wang and Su 2020), another study in ten large Chinese cities reported that the benefits of the emission reduction were partially annulled by adverse meteorology, and severe air pollution events still occurred in the North China Plain (Wang et al. 2020). Sicard et al. (2020) study showed the increase of ozone levels in 4 cities, consistent with the results of the present study. In the Eastern Region of Saudi Arabia, Anil and Alagha (2020) reported similar reductions of NO_2_ (−58%), CO (−13%), and SO_2_ (−9.2%) and the same increase of O_3_ (+17%); however, a different trend was reported for PM_10_ compared to Abu Dhabi results: a decrease of the median PM_10_ was reported (−21%) in Saudi Arabia using data from 7 stations. However, 3 out of these 7 stations reported an increase on the concentrations during lockdown, showing the variability of PM_10_ depending on the location. In Abu Dhabi, an averaged increase of +13% was calculated with all the stations reporting an increase compared to pre-lockdown values.

This study characterises the changes produced in air quality during the lockdown and does not pretend to attribute specifically or quantify the effects of the lockdown since other factors might have influenced the changes, such as weather and regional and long-range transport of pollutants. The air pollution effects of the preventive measures will be a unique opportunity to evaluate the impact of human activities on air quality and assess further air quality policies. After the relaxation of strict preventive measures, an increase of the mobility has been monitored, as shown in this publication, and other commercial and industrial activities, leading to an increase in some of the air pollutants analysed. It is expected that air pollutants will remain low, while the social distancing measures and traffic restrictions are in place. However, there is a risk that countries will design economic stimulus packages to overcome the economic crisis that may result in increased emissions, increased externalities, and continued degradation of nature in the long term. On the other hand, there is also an opportunity to champion solutions that not only rebuild lives and spur economic activity but also accelerate the transition to resilient, low-carbon economies and nature-rich societies (The Club of Rome 2020).

## Conclusion

During the lockdown period aimed to prevent the spread of COVID-19, there has been a significant reduction in the levels of specific air pollutants, especially those related with road traffic, in the Abu Dhabi Region. The concentration of those air pollutants started to increase when the preventive measures were relaxed, although they remained at lower levels than the same period of 2019. This scenario presents a unique opportunity to assess the effects of the reduction of different emission sources. Furthermore, it is a wake-up call to implement green recovery initiatives and social, urban, and mobility policies that maintain reduced air pollution levels and ensure a long-term improvement in air quality and human health.

## Supplementary Information

ESM 1 (PDF 143 kb)

## Data Availability

The data that support the findings of this study are available from the corresponding author, upon reasonable request.
