# Supplementary material for: The influence of COVID-19 preventive measures on the air quality in Abu Dhabi (United Arab Emirates)
Source: Air Qual Atmos Health. 2021 Apr 4;14(7):1071–9. doi: 10.1007/s11869-021-01000-2 (PMC8019479; doi:10.1007/s11869-021-01000-2)
Supplement: Supplementary file 1 — (PDF 143 kb) [file 11869_2021_1000_MOESM1_ESM.pdf]

The influence of COVID-19 preventive measures on the air quality in Abu Dhabi (United Arab Emirates) – SUPPLEMENTARY FILES

Oriol Teixidó (1), Aurelio Tobías (2), Jordi Massagué (2), Ruqaya Mohamed (1), Rasheed Ekaabi (1), Hussein I. Hamed (1), Richard Perry (1), Xavier Querol (2), Shaikha Al Hosani (1).

1. Environment Agency – Abu Dhabi (EAD), United Arab Emirates, Abu Dhabi.
2. Institute of Environmental Assessment and Water Research (IDAEA), Spanish Council for Scientific Research (CSIC), Barcelona, Spain.

Corresponding author at:

Environment Agency – Abu Dhabi (EAD), Al Mamoura Building, Murour Road, PO Box: 45553, Abu Dhabi, United Arab Emirates.

E-mail address: oriol.teixido@ead.gov.ae (Oriol Teixidó)

Supplementary table 1. Environment Agency - Abu Dhabi stations in Abu Dhabi Region.

**Station Name**	**Type**	**SO_2**	**NO_2**	**CO**	**C_6H_6**	**O_3**	**PM_{10}**	**$PM_{2.5}$**
Hamdan Street	Urban Traffic	X	X	X	X	---	X	X
Khadejah School	Urban Background	X	X	---	---	X	X	X
Khalifa School	Suburban Background	X	X	---	---	X	X	X
Al Maqta	Suburban Background	X	X	X	X	X	X	X
Khalifa City A	Suburban Background	X	X	---	---	X	X	X
Baniyas School	Suburban Background	X	X	---	---	X	X	---
Mussafah	Suburban Industrial	X	X	---	---	---	X	X
Al Mafraq	Suburban Industrial	X	X	---	---	---	X	X

Note: (X) Parameter monitored, (---) Parameter not monitored.

Supplementary table 2. Average and p-value of meteorological parameters and mobility indicator by study period in Abu Dhabi, UAE.

	Pre-lockdown	Lockdown		Post-lockdown	
	**Average**	**Average**	**p-value**	**Average**	**p-value**
**Meteorological parameters**					
Wind speed (m/s)	1.9	2.1	0.029	2.0	0.254
Temperature (˚C)	21.0	31.0	<0.001	34.7	<0.001
Net radiation (W/m^2)	133.5	151.0	<0.001	151.3	<0.001
**Mobility Indicator - COVID-19 Community Mobility Reports**					
Average	-2.3%	-36.2%	<0.001	-22.9%	<0.001
Retail & recreational	-5.5%	-49.5%	<0.001	-24.4%	<0.001
Grocery & pharmacy	5.1%	-24.0%	<0.001	-8.5%	<0.001
Parks	-4.7%	-56.5%	<0.001	-37.0%	<0.001
Transit stations	-11.3%	-71.7%	<0.001	-59.6%	<0.001
Workplaces	-0.7%	-38.0%	<0.001	-19.7%	<0.001
Residential	3.1%	22.4%	<0.001	11.6%	<0.001
